# Successful Pedal Bypass in a Patient With Pseudoxanthoma Elasticum

**DOI:** 10.1177/15385744241290007

**Published:** 2024-10-13

**Authors:** Suvi Väärämäki, Olli Hautero, Vesa Rajala, Pasi Nevalainen

**Affiliations:** 1 Centre for Vascular Surgery, 60670 Tampere University Hospital, Faculty of Medicine and Life Sciences, Tampere, Finland; 2 Department of Vascular Surgery, 60656 Vaasa Central Hospital, Vaasa, Finland; 3 Department of Internal Medicine, 60670 Tampere University Hospital, Faculty of Medicine and Life Sciences, Tampere, Finland

**Keywords:** pseudoxanthoma elasticum, critical ischemia, peripheral artery disease, surgical repair, pedal bypass, distal bypass

## Abstract

**Objectives:**

Pseudoxanthoma elasticum (PXE) is a rare metabolic disease, causing calcification in the arterial media layer and further peripheral artery disease (PAD). A high rate of failure has been reported after endovascular and open surgical management of PAD among patients with PXE. Critical limb ischemia (CLI) rarely develops in PXE, and there are only few reports of its treatment.

**Methods:**

We present a case report of a 57 year-old female diagnosed with pseudoxanthoma elasticum (PXE). She presented with critical limb ischemia (CLI) and was successfully treated with pedal bypass using the great saphenous vein.

**Results:**

Despite obtaining suboptimal outcomes through the initial approach of percutaneous transluminal angioplasty to treat critical limb ischemia, the subsequent bypass operation proved to be a success. At the first follow-up appointment at 1 month, the patient was asymptomatic and the ulceration had almost healed. The patient underwent an ultrasound examination at 3, 6, 12, and 24 months after discharge, and the surveillance was uncomplicated.

**Conclusions:**

With a clear indication for surgery, limb-threatening ischemia can be successfully treated with distal bypass, if necessary, in patients with PXE similarly to atherosclerotic PADs. Appropriate diagnostic and surveillance imaging and the utilization of a multidisciplinary team are key components for effective management of PAD in patients with PXE.

## Introduction

Arterial calcification can occur in either the intimal or the medial layer of the arterial wall. Intimal calcification is called atherosclerosis, whereas arteriosclerosis refers to the calcification of the medial layers that is associated with aging or metabolic diseases, such as diabetes and renal insufficiency. Intimal and medial calcification are both associated with peripheral artery disease (PAD), which is a strong independent predictor of cardiovascular mortality.^1^

Arterial calcifications are a notable feature of several genetic diseases, revealing the pivotal role of imbalanced anti-calcifying and pro-calcifying factors, as well as defects in essential key components. Pseudoxanthoma elasticum (PXE) is a rare metabolic disease caused by pathogenic biallelic variants in the ABCC6 gene, with autosomal recessive inheritance. An estimated prevalenceof PXE is around 1:25 000-50 000 people in the general population.^2^ PXE is characterized by elastic fiber fragmentation and calcification in various soft conjunctive tissues, including the skin, eyes, and the arterial media layer.

A diagnosis of PXE requires collaboration between different specialties because it is a suitable combination of skin, retinal and vascular findings, but the genetic testing is the key to diagnosis.^3^ Currently, PXE is incurable, but various preventive treatments have been suggested^4-6^. Current management of PXE is centred on aggressive risk factor control. The ophthalmological manifestations of PXE are the most serious, since they can lead to blindness in late stage of the disease. Intravitreal treatment with vascular endothelial growth factor (VEGF) inhibitors is an effective treatment for stopping choroidal neovascularization and preventing central visual loss.

Calcification of the arterial system is commonly reported already from the third-4^th^ decade of life, without known cardiovascular risk factors.^7^ PAD is found up to 80% of PXE patients and even patients <40 years of age PAD can be diagnosed in 35%^8-11^. Primarily, small- and medium-sized arteries are affected. The pathophysiological process is different in patients with PXE than in common, multifactorial PAD. Thus, questions have been raised about the conventional treatment of PAD among patients with PXE. In addition, a high failure rate has been reported after endovascular and open surgical management of PAD among patients with PXE (Table 1). The mechanism of postoperative failure is unknown. Furthermore, antiplatelet agents are not routinely recommended due to the risk of gastrointestinal mucosal hemorrhage, which is associated with PXE due to the degeneration of the elastic lamina in small arteries.

**Table 1.** Studies Reporting the Results of Endovascular and Open Surgical Interventions Among PXE Patients.

Study	Number of patients (n)	Age (years, median)	Claudication/ CLI (n)	Total number of interventions (n)	Endovascular intervention (n)	Restenosis, occlusion (n)	Bypass surgery (n)	Restenosis, occlusion (n)	Other vascular intervention (n)	Restenosis, occlusion (n)
Verwer^9^	17	59	13/4	58	28	18	12	17	18	-
Ammi^17^	4	55	4/0	15	12	12	3	0	-	-
Siskos^18^	1	58	1/0	1	1	0	-	-	-	-
Donas^19^	1	51	1/0	1	1	0	-	-	-	-
Väärämäki^5^	1	59	0/1	1	-	-	-	-	1	0

The typical symptom of PAD is claudication. A first-line treatment for claudication is walking exercise. In critical limb ischemia (CLI), defined by ischemic pain at rest or ischemic tissue loss, the first-line treatment is revascularization. The progression of arteriosclerosis in PXE occurs at a comparably slower rate than intimal atherosclerosis. Consequently, critical limb ischemia rarely develops in PXE, leading to a lesser demand for revascularization. Notably, PXE patients are usually younger than typical PAD patients. They are physically active when the disease starts to develop, which promotes robust development of collateral circulation, resulting in minor or even absent symptoms.

PXE is a very rare disease for a vascular surgeon to come across, and most of the current reports are small cohorts and case reports. The largest retrospective study reported a 50.8% prevalence of PAD among PXE patients, most of whom were treated conservatively.^9^ There are no reports of ischemic wounds nor of successful pedal bypass in patients with PXE. Only a solitary case report documents an ischemic wound in a PXE-like disease.

## Case Report

A 57 year-old female who was on medication for hypertension, hypercholesterolemia, and *LADA* (latent autoimmune *diabetes* in adults) was referred to a vascular surgeon because of an ulceration in the left lower limb. She had never suffered from claudication, but rest pain has developed in the left limb over the last 2 weeks. She had never smoked. At the age of 56, she had had an intracerebral hemorrhage (ICH) without findings of intracranial aneurysms or arterial malformations. Pseudoxanthoma elasticum (PXE) had been diagnosed previously at the age of 44 based on central vision loss, typical angioid streaks in the retina, and typical skin lesions on the sides of the neck. The diagnosis was further confirmed by typical findings in the skin biopsy and later by genetic testing (ABCC6 homozygous c.3421 C>T, p.[Arg1141Ter]). A decreased ankle-brachial index (ABI; right 0.77and left 0.62) was already noted at the time of diagnosis, without symptoms.

**Figure 1.**
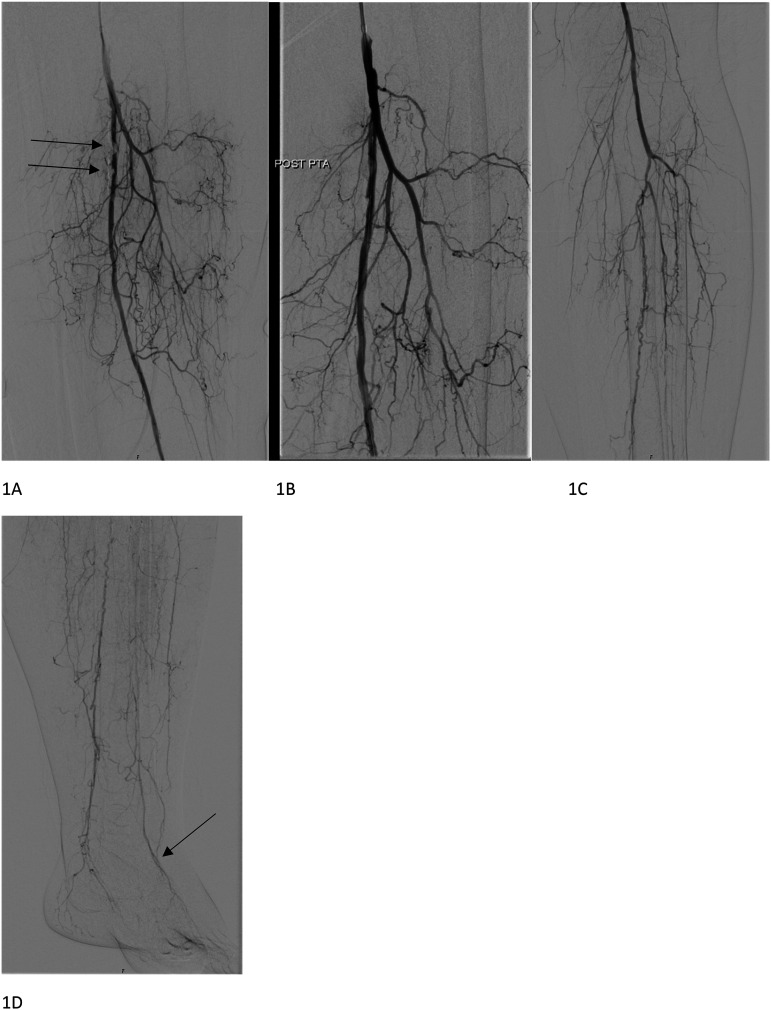
Stenosis of the left superficial femoral artery (SFA) before (A) and after (B) percutaneous transluminal angioplasty (PTA). Stenosed segments in the infrapopliteal arteries (C–D) and the target artery (pedal artery [arrow]) for pedal bypass (D).

At the vascular surgery outpatient clinic, ABI at rest was 0.57 on the right and 0.46 on the left side. Angiography showed stenosis of the left superficial femoral artery (SFA), as well as several stenosed segments in the infrapopliteal arteries (Figure 1). A standard percutaneous transluminal angioplasty (PTA) was performed in the SFA with a good angiologic result (Figure 1(B)). Clopidogrel- therapy was started, but dual therapy (aspirin and clopidogrel) was avoided.

**Figure 2.**
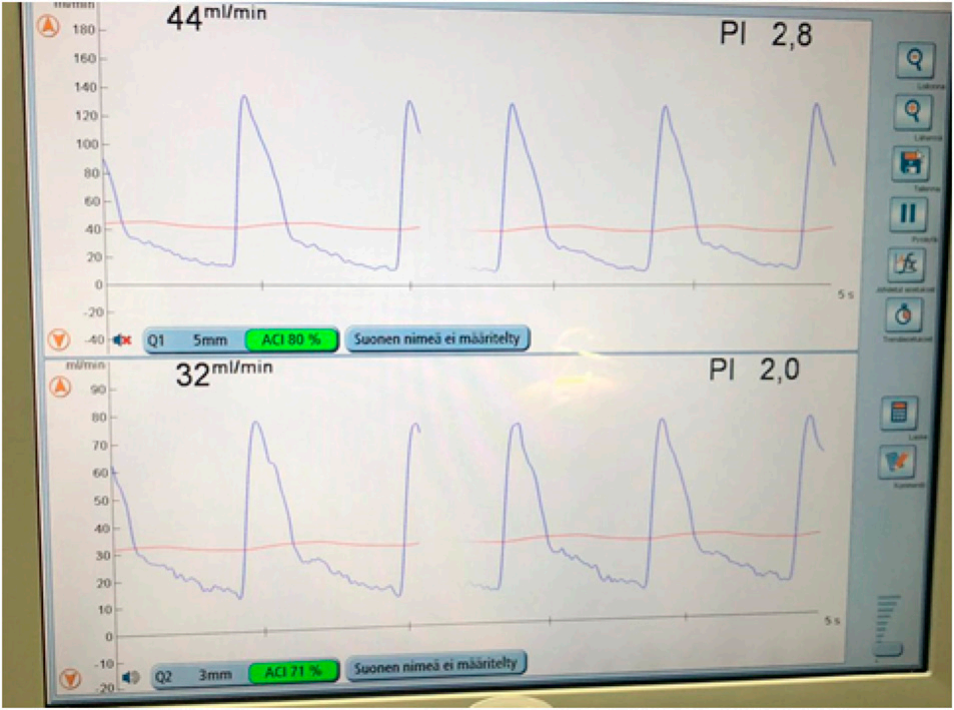
Volume flow measurement with a perivascular flowmeter from the proximal (top) and distal part of the pedal vein graft (bottom).

At 1 month, the rest pain had been relieved, but the ulceration was mildly infected. ABI at rest was 0.44 on the left side. The patient had had no bleeding complications due to antiplatelet therapy. The clinical outcome of the PTA was poor, and revascularization was required. Pedal bypass from the common femoral artery to the dorsal artery of the foot was performed in situ 1 month later using the great saphenous vein (Figure 2).

The surgical procedure and recovery were uncomplicated. At the 1-month control visit, the operated left limb was asymptomatic, and the ulceration had almost healed. The toe pressure was 59 mmHg, and low-dose aspirin treatment (100 mg per day) was continued as permanent medication, since the patient had no history of gastrointestinal bleeding.

Duplex ultrasound (DUS) surveillance of the bypass graft was carried out at 3, 6, 12, and 24 months postoperatively. The DUS surveillance was uncomplicated and was therefore concluded at 2 years. The ABI was 0.66 on the right and 0.96 on the left leg and toe pressures 45mmHg (right) and 53 mmHg (left) at 2 years. So far, the operated limb has remained asymptomatic for 3 years. The patient has tolerated antiplatelet monotherapy well.

## Discussion

In instances of peripheral artery disease at a young age, particularly in the absence of evident cardiovascular risk factors, it is imperative to consistently consider the possibility of a rare condition like PXE. As is common, our patient was diagnosed with PXE by a dermatologist and ophthalmologist based on pseudoxanthomas of the skin, with typical biopsy findings and retinal angioid streaks, respectively. Our patient already had a decreased ABI at the time of the diagnosis, but she was asymptomatic, probably due to collateral vasculature developed by physical activity.

An increased risk of hemorrhage has been reported in PXE, and especially gastrointestinal hemorrhage is a life-threatening condition.^12,13^ Our patient had previously received a diagnosis of an intracerebral hemorrhage (ICH) with no apparent cause. The pathophysiologic mechanism of the hemorrhagic tendency remains elusive; nonetheless, a hypothesis has been proposed implicating the disintegration of elastic fibers as a potential trigger for an arterial wall rupture.^13^ Furthermore, an increased risk of vascular malformations is suggested to be a novel feature among patients with PXE.^14^ Our patient was treated with aspirin as well as clopidogrel dispite of earlier ICH and the dual antiplatelet therapy was uncomplicated.

The development of CLI in a patient with PXE is an infrequent occurrence, leading to a scarcity of available data regarding its optimal treatment approach. There are data suggesting a high and early failure rate after vascular interventions (Table 1). Open surgery has been proven to be more effective than endovascular therapy in the treatment of limb-threatening ischemia in infrainguinal PAD if the patient has an adequate great saphenous vein for surgical revascularization and it could be considered even the first line therapy.^15^ Also, reported patency rates of great saphenous vein graft at 1 and 2 years are high (primary 87%, 78%).^16^ Based on our case, open surgical repair is also a preferable approach in limb-threatening ischemia in PXE.

Presently, there are no established standards for either screening or monitoring the potential complications during follow-up. No reinterventions were required for our patient during a standard DUS surveillance of the bypass graft. To date, no treatment is able to halt the progression of calcification, which would be key for the treatment of PXE. This case highlights the necessity of clinical collaboration between various specialties to reach a diagnosis and of multidisciplinary co-operation during follow-up in this rare disease. As PXE is systemic metabolic disease, there is a wide variability in the presentation of the affected organs and therefore its important to refer these patients also for other specialists.

## Conclusion

Based on our experience, limb-threatening ischemia can be treated successfully with distal bypass, if necessary, in patients with PXE, similarly to common atherosclerotic PAD. Low-dose antiplatelet therapy can be used safely after careful consideration if the patient has no history of gastrointestinal bleeding.
